# A comparison of bee communities between primary and mature secondary forests in the longleaf pine ecosystem

**DOI:** 10.1038/s41598-020-59878-4

**Published:** 2020-02-19

**Authors:** Michael D. Ulyshen, Scott Pokswinski, J. Kevin Hiers

**Affiliations:** 1 USDA Forest Service, Southern Research Station, Athens, 320 Green Street, Athens, Georgia 30602 USA; 2 Tall Timbers Research Station, 13093 Henry Beadel Dr., Tallahassee, Florida 32312 USA

**Keywords:** Ecology, Zoology

## Abstract

Much of the once-dominant longleaf pine (*Pinus palustris* Mill.) ecosystem has been lost from the Coastal Plain of the southeastern United States and only a few scattered remnants of primary forest remain. Despite much interest in understanding and restoring this ecosystem, relatively few studies have attempted to characterize or assess the conservation status of the longleaf bee fauna. The objective of this study was to compare the diversity and composition of bee communities between primary and mature secondary (>100 years old) fire-maintained forests in Georgia and Florida. We used colored pan traps to sample bees at three primary and four secondary locations divided between two regions characterized by sandy (Eglin Air Force Base) or clayey (Red Hills) soils. There were no overall differences between primary and secondary forests in bee richness, diversity, evenness or abundance. Community composition differed among locations but we found no evidence that primary remnants provide critical habitat to sensitive bee species.

## Introduction

Natural habitats play a key role in supporting diverse pollinator populations (i.e., bees, flies, butterflies, etc.) and the conversion of these areas to intensive agriculture has been identified as one of the major drivers of pollinator declines^1,2^. Efforts to understand bee diversity in natural habitats and how these organisms are affected by disturbance history and management decisions are therefore of great interest. Although the value of forests to pollinators has received relatively little attention compared to other land use categories, it is clear that forest type, forest age, disturbance history, management practices and other factors can strongly influence the diversity of bees and other flower-visiting insects^3,4^. However, much remains unknown about how pollinator communities change as forests age or how these assemblages differ between primary and secondary forests. This kind of descriptive information is essential for assessing the status and recovery of pollinator communities within a given forest type following harvest or canopy loss. Indeed, a recent gathering of stakeholders identified a shortage of baseline information about pollinators in managed conifer forests as one of the biggest knowledge gaps facing land managers and long term ecosystem recovery^5^.

Primary forests, defined as those that have experienced little or no known anthropogenic disturbance^6^, provide a special opportunity to assess the diversity and composition of biotic communities under minimal human influence. Although there is strong support for the conclusion that primary or old-growth forests support specific taxa that are rare or absent elsewhere^7^, studies comparing bee biodiversity between primary and secondary forests have yielded mixed results. Some have reported higher diversity in primary forests^8^, while others have found either no significant differences^9^ or that diversity is higher in secondary forests^10,11^. The relative importance of primary and secondary forests to bees may vary with forest type due to differences in forest structure and patterns of disturbance. For example, light transmittance through the canopy is known to be an important factor for both bees and herbaceous plant availability and can either decrease or increase with forest age depending on the forest type. By contrast to the closed canopy conditions of primary rainforests, for instance, conifer forests maintained by frequent fires generally become more open with time to form savanna-like habitats with a diverse understory plant community^12^.

The purpose of the current study was to compare the diversity and composition of bee communities between primary and mature secondary longleaf pine (*Pinus palustris* Mill.) forests in the southeastern United States. Beginning about 7500–5000 ybp, the longleaf pine ecosystem once covered much of the southeastern U.S. coastal plain, extending from Virginia to Texas, but is now considered among the most endangered ecosystems in North America^13–15^. It currently occupies about 2% of its historic range and only about 0.01% of primary forests remain, amounting to about 5100 ha in total^16–18^. Primary longleaf pine ecosystems are characterized by widely spaced trees (dominated by *P. palustris*) in the overstory and extremely diverse herbaceous plant communities in the understory^19^. Plant communities vary with soil type but diversity commonly exceeds 40 species per square meter and can reach as high as 140 species per 1000 m^2^ ^20^. Frequent fires are required to maintain the open conditions and high diversity characteristic of this ecosystem, and plant flowering following fire has been hypothesized to be important to pollinators^12,21^.

Approximately 75% of plant species endemic to the longleaf pine ecosystem depend on insects for pollination and most of these species are visited by multiple species of bees^22^. Fire dependent groups, such as paplionoid legumes, are common to these ecosystems and are pollinated almost exclusively by bees with a variety of responses to fire-altered phenology^12^. Several previous studies have sampled bees in longleaf pine forests^23–27^ but no effort, to our knowledge, has been made to determine how bee assemblages differ between primary and secondary forests. Breland *et al*.^23^ recently compared bee communities between secondary longleaf pine forests with and without a history of tillage agriculture but otherwise very little is known about how bees are affected by past disturbances.

In this study, we sampled bees in three of the most pristine primary longleaf pine remnants as well as in four mature secondary forests located nearby. Because plant communities are strongly determined by soil type, our locations were divided between two regions characterized by either sandy or clayey soils. We hypothesized that bee diversity and the abundance of sensitive species would be significantly higher in the primary forests than in the mature secondary forests.

## Methods

### Study locations

**Figure 1:**
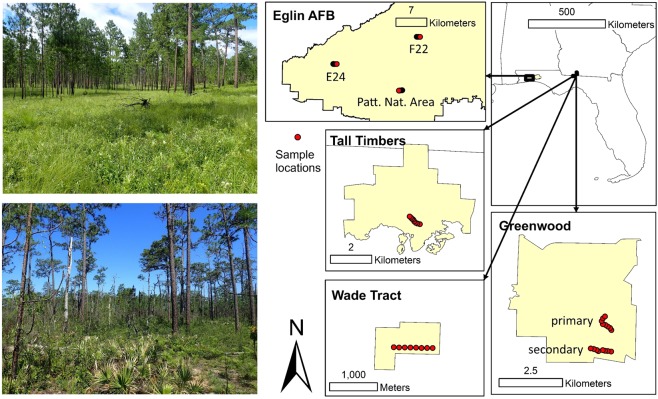
Pictures of primary forests used in this study and a map of the study areas. The top picture is from the Wade Tract in the Red Hills and the bottom picture is from Patterson Natural area on Eglin Airforce Base. Bees were sampled in eight plots (red circles) at each location. The map was created using ArcMap 10.5.1 (https://www.esri.com).

Bees were sampled at seven locations on the southeastern U.S. Coastal Plain, all within the historic range of longleaf pine (Table 1). The locations were divided between Eglin Airforce Base (Okaloosa and Santa Rosa Counties, FL) and the Red Hills region near the border of Georgia and Florida. Although Eglin AFB receives more rainfall than the Red Hills, productivity is higher in the Red Hills due to higher quality soils (i.e., clayey ultisols vs. xeric sandy quartzipsamments)^16,28,29^. These two regions contain some of the best remaining primary longleaf pine remnants, >70% of which occur at Eglin AFB alone. At Eglin AFB, we selected one primary forest (Patterson Natural Area) and two mature secondary forests (E24 and F22), separated by at least 10 km (Fig. 1). Within the Red Hills, we selected two primary forest locations (Greenwood and the Wade Tract) and two mature secondary forests (Greenwood Secondary and Tall Timbers Research Station) (Fig. 1). The Wade Tract and two Greenwood locations are privately owned properties in Thomas County, Georgia. The primary and secondary Greenwood locations were separated by about 1 km while the distance between the Greenwood locations and the Wade Tract was about 8–9 km. Tall Timbers Research Station is located in Florida about 25–30 km away from the other Red Hills locations. It is the only location in this study that is not dominated by longleaf pine in the overstory, being instead dominated by loblolly pine. Despite this difference, stand conditions and management history are similar to the secondary longleaf pine forests of the region.

**Table 1:** Information for the seven locations sampled in this study.

Region	Location	Coordinates	Age	Stand size (ha)	Forest age (years)	Basal area (mean ± SE m^2^/ha)	Fire frequency (return interval, years)	Known Soil disturbance history
Eglin AFB	Patterson Natural Area	30.487154–86.741373	Primary	2031	400+	5.3 ± 1.1	2	None
	E-24	30.540883–86.875927	Secondary	~650	125	7.7 ± 1.0	2.5	None
	F-22	30.596333–86.706026	Secondary	~500	125	6.8 ± 0.6	2	None
Red Hills	Wade Tract	30.758367–83.999040	Primary	83	350+	14.2 ± 2.9	2	None
	Tall Timbers	30.651599–84.226949	Secondary	~150	125	8.1 ± 2.0	1.5	tilled until 1890
	Greenwood Secondary	30.836033–84.018704	Secondary	~250	100	10.6 ± 2.2	2	None
	Greenwood Primary	30.844923–84.017743	Primary	200	300+	13.4 ± 2.1	2	None

The sizes of primary forest remnants were taken from Varner and Kush^17^ and basal areas are from Ulyshen, *et al*.^55^. Forest ages refer to the approximate ages of the living trees and fire frequency is based on the ten-year period from 2008–2017.

All seven locations sampled in this study had been burned regularly (i.e., every ~1.5–2.5 yrs, see Table 1) for at least several decades to maintain the open conditions characteristic of the longleaf pine ecosystem. The Red Hills locations had all been burned on this schedule since the 1920s whereas the locations at Eglin AFB underwent a period of infrequent fire from the 1950s–1980s before prescribed fire was again implemented as part of conservation management of the Red-Cockaded Woodpecker^30^. Among the four secondary locations, only Tall Timbers had experienced major soil disturbance, with a legacy of agriculture from the 1800 s. Tall Timbers was thus characterized by “old-field” vegetation common to second growth forests where species are largely native but have lost the characteristic bunch grasses of untilled understory communities. The secondary site at Greenwood was dominated by longleaf and slash pine overstory and retained understory characteristics of sites that had never been tilled following clearcutting in the early 20^th^ century. The secondary locations at Eglin AFB had never been tilled and the understory was dominated by *Schizachyrium scoparium* (Michx.) Nash and other native bunchgrasses.

### Bee sampling and data collection

We established eight sampling plots at each location (i.e., 56 plots in total), separated by 100 m along a 700 m linear transect. The 100 m spacing between sampling plots was selected so that a complete transect could fit within the boundaries of the Wade Tract, the smallest forest sampled in this study (Fig. 1). Bees were sampled using a set of three pan traps (white, yellow and blue) at each sampling plot. Pan traps represent an effective and highly standardized method for sample pollinator communities but are known to be more effective at capturing some bee taxa (e.g., halictids) than others (e.g., bumblebees)^31,32^. Although the pan traps likely yielded an incomplete picture of the bee community at each location, this approach allowed us to sample bees simultaneously and consistently at all 56 plots. The traps consisted of plastic food bowls that were suspended ~20 cm above the ground on wire stands. The three traps at each plot were arranged in a row following the direction of the transect and were separated from one another by 5 m. We attempted to sample bees throughout the 2017 growing season at all locations in the Red Hills and on Eglin AFB. Due to access difficulties, however, we were only able to sample twice at all three Eglin AFB locations (20–23 Mar and 17–24 October), for a total of ten sampling days. Results presented here for Eglin AFB are thus limited to these periods. By contrast, there were a total of seven sampling periods at the Red Hills locations (10–13 March, 4–7 April, 2–5 May, 30 May- 2 June, 28 June- 1 July, 25–28 July and 1–4 October), for a total of 21 sampling days. Because of these large differences in sampling intensity and timing, the results from Eglin AFB and Red Hills are analyzed and presented separately. Bee specimens were identified to species using both printed^33–36^ and online resources (discoverlife.org).

### Analysis

We calculated the total richness (i.e., number of species), Shannon’s diversity index, evenness and total abundance of bees captured at each sampling plot after pooling across sampling dates. To compare how these bee metrics varied among locations, we performed analysis of variance (function aov) using R 3.6.1^37^ with location and canopy openness (covariate) included as predictors in all models. All metrics satisfied normality assumptions. Pairwise comparisons were based on differences in LS means (using the lsmeans package) with the Tukey-adjusted significance level^38^. A separate contrast was performed to specifically compare between primary and secondary forests for each of these metrics. To assess how bee communities differed among Red Hills locations, we performed nonmetric multidimensional scaling using PC-ORD ver. 6^39^. Only bee species that were captured in at least three sampling plots were included in this analysis and data were relativized by species maximum prior to the analysis. The same analysis was attempted for the Eglin AFB locations but a useful NMDS ordination was not found. We then used PERMANOVA with pairwise comparisons for the Red Hill and Eglin AFB data to test whether community composition varied among locations. Finally, we tested whether any species were strongly associated with one or several locations using the function multipatt (multilevel pattern analysis) in the R package indicspecies^40^. This approach goes beyond the traditional indicator species analysis developed by Dufrêne and Legendre^41^ by testing for associations with combinations of groups in addition to associations with specific groups. Indicator values range from 0 (no association) to 1 (complete association).

## Results

A total of 94 bee species from 5,869 individuals were collected, with 78 and 39 species found in the Red Hills and Eglin AFB locations, respectively (Supplementary Table S1). The three most common species at Eglin AFB were *Lasioglossum apopkense* (Robertson) (63.5%), *L. nymphale* (Smith) (13.5%) and *L. illinoense* (Robertson) (9.8%). The three most common species from the Red Hills were *L. apopkense* (Robertson) (16.2%)*, L. reticulatum* (Robertson) (15.3%) and *Augochlorella aurata* (Smith) (14.5%).

**Figure 2:**
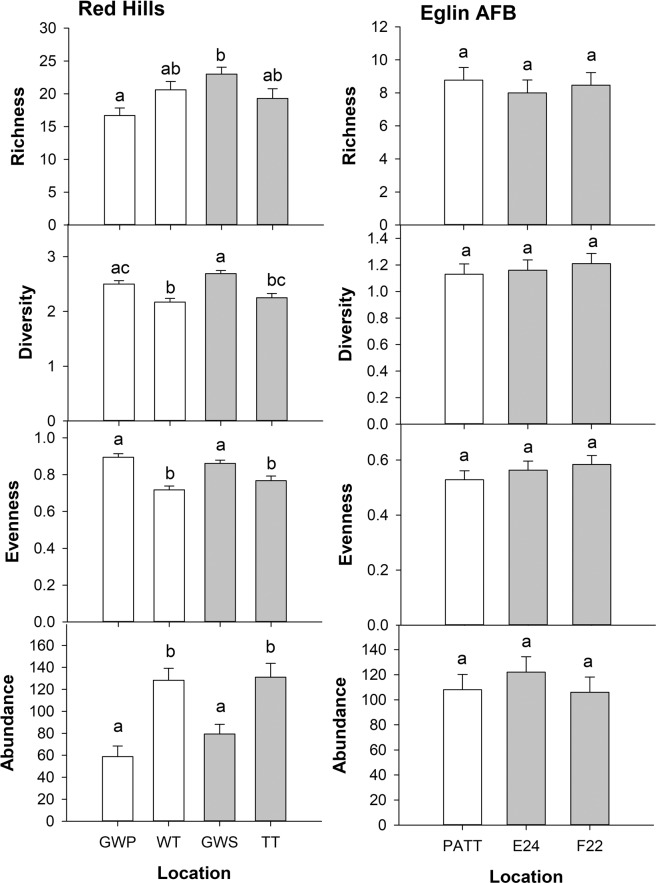
Least square means ± SE (n = 8) bee richness, diversity and abundance for locations in the Red Hills (left) and at Eglin Airforce Base (right). White and grey bars represent primary and secondary locations, respectively. Bars with different letters above them are significantly different.

Bee richness (F_3,27_ = 8.6, P < 0.001), diversity (F_3,27_ = 20.5, P < 0.0001), evenness (F_3,27_ = 21.6, P < 0.0001) and abundance (F_3,27_ = 22.9, P < 0.0001) varied significantly among the four Red Hills locations (Fig. 2). Based on differences in LS means, bee richness was higher at Greenwood secondary than at the Greenwood primary location (Fig. 2). Bee diversity was significantly higher at the Greenwood secondary location than at Tall Timbers or the Wade Tract and bee evenness was higher at both Greenwood locations than at the other two Red Hills locations (Fig. 2). Bee abundance was significantly higher at Tall Timbers and at the Wade Tract than at the other two locations (Fig. 2). There were no differences overall between primary and secondary locations in the Red Hills for bee richness (F_1,27_ = 2.5, P = 0.1), diversity (F_1,27_ = 2.9, P = 0.1), evenness (F_1,27_ = 0.1, P = 0.8) or abundance (F_1,27_ = 0.8, P = 0.4). There were no differences in bee richness (F_2,20_ = 0.4, P = 0.6), diversity (F_2,20_ = 0.3, P = 0.7), evenness (F_2,20_ = 0.9, P = 0.4) or abundance (F_2,20_ = 0.1, P = 0.9) among the three Eglin AFB locations (Fig. 2). Similarly, there were no differences between primary and secondary forests at Eglin AFB for richness (F_1,20_ = 0.3, P = 0.6), diversity (F_1,20_ = 0.4, P = 0.6), evenness (F_1,20_ = 1.2, P = 0.3) or abundance (F_1,20_ = 0.2, P = 0.7).

**Figure 3:**
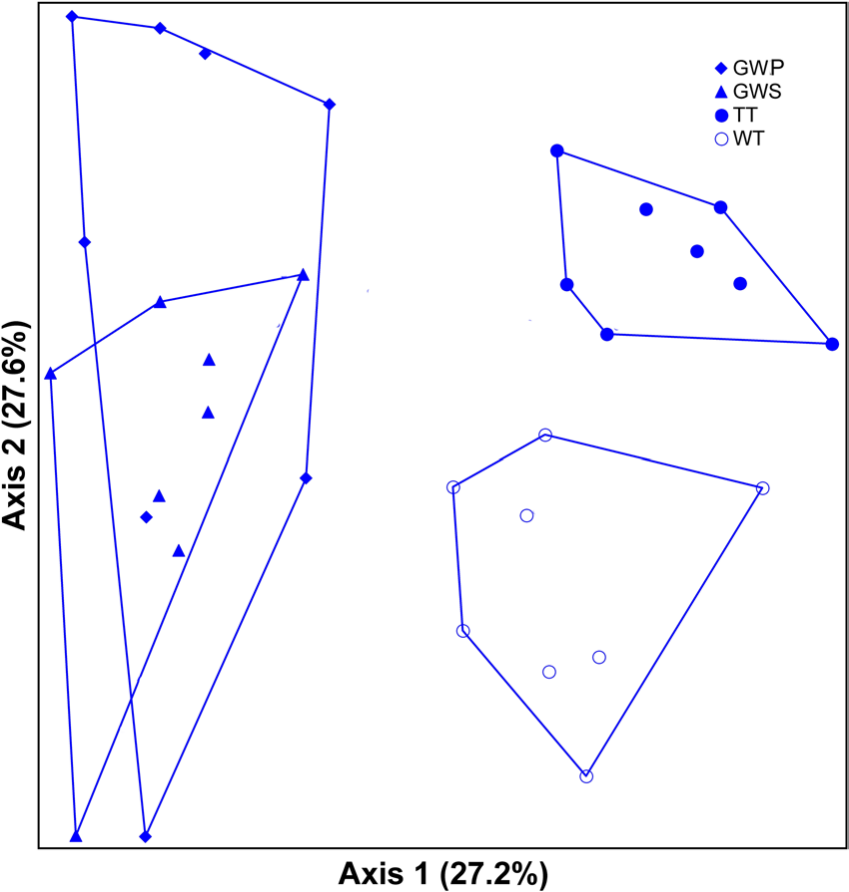
Ordination from non-metric multidimensional scaling showing differences in bee composition among Red Hills locations. Abbreviations are as follows: TT = Tall Timbers (secondary); GWS = Greenwood Secondary; GWP = Greenwood Primary and WT = Wade Tract (primary).

Non-metric multidimensional scaling yielded a three-dimensional solution with a final stress of 17.65. It is clear from the ordination that bee community composition varied greatly among the Red Hills locations (Fig. 3). The samples from Tall Timbers and the Wade Tract form distinct groupings and are also widely divergent from the Greenwood samples. There is more overlap between the Greenwood primary and secondary locations but composition differed significantly among all four Red Hills locations based on PERMANOVA analysis (Supplementary Table S2). PERMANOVA also detected significant differences in bee composition among all three locations at Eglin AFB (Supplementary Table S2).

Based on indicator species analysis, 20 species captured at the Red Hills locations were strongly associated with one or several of the four locations (Table 2). Ten species were associated with one location, seven with two locations and three with three locations. Only two of these species were found to be strongly associated primary forests (*Andrena violae* Robertson and *Lasioglossum vierecki* (Crawford)) and both of these are common and widespread taxa. Among the Eglin AFB locations, six species were significantly associated with one location (Table 3). Only one of these species, *Lasioglossum trigeminum* Gibbs, also a common and widespread species, was significantly associated with the primary site.

**Table 2:** Bee species significantly associated with one or more locations in the Red Hills region based on indicspecies analysis.

Species	GWP	GWS	TT	WT	statistic
*Andrena violae* Robertson	x				0.567, P = 0.049
*Anthophorula micheneri* Timberlake		x			0.612, P = 0.042
*Augochlorella aurata* (Smith)	x		x	x	0.945, P = 0.007
*Augochloropsis anonyma* (Cockerell)	x		x		0.866, P = 0.004
*Augochloropsis metallica* (Fabricius)			x	x	0.918, P = 0.001
*Augochloropsis sumptuosa* (Smith)			x	x	0.968, P = 0.001
*Ceratina dupla* Say			x		0.685, P = 0.017
*Ceratina floridana* Mitchell			x		0.973, P = 0.001
*Eucera dubitata* (Cresson)			x		0.822, P = 0.003
*Halictus ligatus* Say			x	x	0.789, P = 0.007
*Lasioglossum (D.) batya* Gibbs		x		x	0.866, P = 0.001
*Lasioglossum (D.) floridanum* (Robertson)		x		x	0.707, P = 0.013
*Lasioglossum (D.) hitchensi* Gibbs		x			0.791, P = 0.004
*Lasioglossum (D.) longifrons* (Baker)	x	x			0.84, P = 0.002
*Lasioglossum (H.) nelumbonis* (Robertson)			x		0.832, P = 0.002
*Lasioglossum (D.) vierecki* (Crawford)				x	0.757, P = 0.005
*Melissodes communis* Cresson		x	x	x	0.876, P = 0.026
*Melissodes comptoides* Robertson			x		0.791, P = 0.002
*Perdita gerardiae* Crawford	x	x		x	0.945, P = 0.007
*Svastra atripes* (Cresson)		x			0.645, P = 0.04

**Table 3:** Bee species significantly associated with one or more locations at Eglin Air Force Base based on indicspecies analysis.

Species	E24	F22	PATT	statistic
*Lasioglossum (D.) reticulatum* (Robertson)	x			0.932, P = 0.001
*Lasioglossum (D.) trigeminum* Gibbs			x	0.791, P = 0.003
*Osmia sandhouseae* Mitchell		x		0.707, P = 0.02
*Perdita georgica* Timberlake		x		0.707, P = 0.021
*Perdita gerardiae* Crawford	x			0.707, P = 0.016
*Svastra atripes* (Cresson)	x			0.866, P = 0.002

## Discussion

This study sought to establish a baseline understanding of bee communities native to the longleaf pine ecosystem by making comparisons between primary and mature secondary forests. Overall, we found no significant differences in bee richness, diversity or abundance between these forest types. Moreover, we also found no evidence that primary forest relicts support distinct communities or notably large populations of sensitive bee taxa. These results are consistent with Breland *et al*.^23^, the one previous study to compare bee communities between longleaf pine forests with differing disturbance histories. In that study, bee communities did not differ between small adjacent plots that had or had not been previously subjected to tillage agriculture. Interestingly, plants were strongly affected by agricultural history in that same study^42,43^, suggesting that local responses of plants do not necessarily extend to the highly mobile bee community. Such findings may also explain the diverse pollinator syndromes found across bee pollinated taxa and lack of sensitivity to changes in plant phenology^12^.

Previous studies suggest bees may be more resilient to anthropogenic forest disturbance than other taxa. In Brazil, for example, Barlow *et al*.^7^ compared the diversity of 15 plant and animal taxa among primary rainforest, secondary forests and non-native *Eucalyptus* plantations. While many of the groups were clearly more diverse in primary forests, this was not the case for euglossine bees. Almost all species found in primary forests were also found in secondary forests and most of them were also found in the *Eucalyptus* plantations. A more recent analysis of the same data found that euglossine bees were the only taxonomic group found not to be nested according to land use intensity, i.e., bees in disturbed forests were not simply subsets of species found in primary forests^44^. These patterns may be driven by the strong flight capabilities of euglossine bees^45^ which exceed those of many other bee taxa^46^. Studies in other parts of the world have documented stronger effects of disturbance on bee diversity. In China, for example, Hua *et al*.^47^ found bee diversity to be much higher in native forests than in *Eucalyptus*, bamboo, Japanese cedar or mixed forests planted on abandoned crop land. Similarly, Taki *et al*.^8^ reported higher bee richness from primary than secondary forests in Japan.

While our results indicate that mature secondary open pine forests maintained with frequent fire support a diversity of bees comparable to that of primary longleaf pine forests, it is important to note that the secondary forests used in this study were themselves mature at >100 years old. It is possible that comparisons among significantly younger forests would show greater short-term differences among bee communities. Nevertheless, the secondary forests sampled in this study are highly representative of lands managed for longleaf pine restoration^48,49^ and our results suggest that bees are resilient over the long-term to past disturbances.

This resilience has been documented in other highly altered southern landscapes where fire and thinning result in more open stands and support a diverse understory flora. Hanula *et al*.^3^ compared bee communities among different forest types in central Georgia and found mature open loblolly pine (*P. taeda* L.) forests similar to those used in this study to yield significantly higher bee abundance and richness on average than dense young pine stands or thinned young pine stands. Although that study did not involve longleaf pine forests, the findings suggest that bee habitat quality may increase with time in fire-maintained pine forests of the southeastern United States. This pattern may be driven by canopy openness as the open stand conditions created by frequent fires are likely to benefit bees by increasing floral resource availability near the forest floor. Indeed, experimental thinning of longleaf pine forests in South Carolina resulted in significant increases in plant cover and richness as well as in bee abundance and richness^23^.

Colored pan traps provide a reliable and standardized method for sampling bees but are known to capture only a portion of the locally active bee fauna^31,32^. Our results may therefore underestimate the diversity of bees present at our locations. Bartholomew *et al*.^27^ compiled a checklist of 165 bee species known from the longleaf pine savannas of Louisiana and Mississippi and speculated this region may support up to 200 species in total, more than twice the number collected in this study. As reported from other systems^50,51^, our results from the Red Hills indicate a high degree of turnover between even narrowly separated locations, suggesting that the total diversity of bees endemic to the longleaf pine ecosystem may indeed be quite high. Unlike deserts where half or more of species are oligolectic^52,53^, Bartholomew *et al*.^27^ estimated an oligolecty rate of 18% among bees known from the longleaf pine ecosystem. It is therefore not surprising that most of the 94 bee species collected in this study are common and widespread taxa not limited to the longleaf pine ecosystem.

## Conclusions

We detected no significant differences in the diversity or abundance of bees between remnant primary longleaf pine forests and mature secondary forests managed for conservation and restoration. Considering the extent and variety of the longleaf pine ecosystem and the limitations inherent to pan trapping, much more work is needed to fully answer these questions. Approximately 40% of all plant species on the southeastern coastal plain are endemic to the longleaf pine ecosystem. Moreover, many of these plant species (127) as well as a number of vertebrates are endangered or considered threatened^14^. Although no bees were among the ten species of insects Noss and Scott^13^ listed as being at risk of becoming endangered in the longleaf pine ecosystem, the extent to which this reflects the resiliency of bees to forest or soil disturbance, as suggested by the current study, or insufficient attention given to the conservation status of insects^54^, remains to be seen. Our work, however, suggest that longleaf pollinator communities are resilient to disturbance and were little affected by the regional wave of forest harvest and mid 20^th^ century disruption of frequent fire regimes experienced by this ecosystem.

## Supplementary information

Supplementary information.
