# Supplementary material for: A comparison of bee communities between primary and mature secondary forests in the longleaf pine ecosystem
Source: Sci Rep. 2020 Feb 19;10:2916. doi: 10.1038/s41598-020-59878-4 (PMC7031531; doi:10.1038/s41598-020-59878-4)
Supplement: Supplementary file 1 — Supplementary information. [file 41598_2020_59878_MOESM1_ESM.pdf]

Supplementary Information

A comparison of bee communities between primary and mature secondary forests in the longleaf pine ecosystem

Michael D. Ulyshen[1*], Scott Pokswinski[2], and J. Kevin Hiers[2]

Supplementary Table S1. List of bee species and abundance by location. Abbreviations are as follows: PATT=Patterson Natural Area; TT=Tall Timbers; GWS=Greenwood Secondary; GWP=Greenwood Primary and WT=Wade Tract.

	Eglin AFB			Red Hills				
	Secondary		Primary	Secondary		Primary		
Species	E24	F22	PATT	TT	GWS	GWP	WT	Total
Agapostemon splendens (Lepeletier)	1	0	0	0	0	0	0	1
Andrena banksi Malloch	0	0	0	0	1	0	0	1
Andrena barbara Bouseman and LaBerge	0	0	0	0	2	1	0	3
Andrena confederata Viereck	0	0	0	1	0	0	0	1
Andrena cressoni Robertson	0	0	0	0	0	0	1	1
Andrena fenningeri Viereck	0	0	0	0	4	0	0	4
Andrena fulvipennis (Smith)	1	0	0	0	0	0	0	1
Andrena hilaris Smith	0	0	0	0	0	3	1	4
Andrena imitatrix Cresson	0	1	1	1	1	0	0	4
Andrena miserabilis Cresson	0	0	1	0	0	0	0	1
Andrena neonana Viereck	0	0	0	0	1	0	0	1
Andrena perplexa Smith	0	0	0	0	2	0	0	2
Andrena sayi? Robertson	0	0	0	0	0	0	1	1
Andrena violae Robertson	0	0	0	0	0	6	1	7
Anthophorula micheneri Timberlake	0	0	0	0	7	0	0	7
Apis mellifera L.	0	0	1	5	3	1	2	12
Augochlorella aurata (Smith)	0	0	0	372	12	32	46	462
Augochloropsis anonyma (Cockerell)	0	0	2	36	8	24	2	72
Augochloropsis metallica (Fabricius)	0	0	0	37	2	0	14	53
Augochloropsis sumptuosa (Smith)	0	0	3	73	0	0	33	109
Bombus griseocollis (De Geer)	1	0	0	0	1	0	0	2
Bombus impatiens Cresson	0	0	0	4	4	7	6	21
Ceratina cockerelli Smith	0	1	0	0	0	0	0	1

Ceratina dupla Say	0	0	0	6	2	0	0	8
Ceratina floridana Mitchell	0	0	0	89	5	0	0	94
Coelioxys germana Cresson	1	0	0	0	0	0	0	1
Coelioxys sayi Robertson	0	0	1	0	0	0	0	1
Colletes americanus Cresson	0	1	0	0	0	0	0	1
Colletes longifacies Stephen	0	2	0	0	0	0	0	2
Colletes thoracicus Smith	0	1	0	0	0	0	0	1
Colletes sp. 1	0	0	0	1	0	0	0	1
Colletes sp. 2	0	0	0	1	0	0	0	1
Epeolus erigeronis Mitchell	0	0	0	1	0	0	0	1
Eucera dubitata (Cresson)	0	0	0	9	0	0	1	10
Eucera fulvohirta (Cresson)	0	0	0	1	0	1	0	2
Eucera rosae (Robertson)	0	0	0	1	0	0	0	1
Habropoda laboriosa (Fabricius)	1	11	2	0	0	0	0	14
Halictus ligatus Say	0	0	0	22	2	1	7	32
Hylaeus georgicus (Cockerell)	0	0	0	0	0	1	0	1
Lasioglossum (D.) apopkense (Robertson)	562	542	604	97	68	55	295	2223
Lasioglossum (D.) batya Gibbs	3	5	7	0	30	0	13	58
Lasioglossum (D.) bruneri (Crawford)	0	0	0	2	2	0	0	4
Lasioglossum (D.) callidum (Sandhouse)	0	0	0	1	2	1	0	4
Lasioglossum (E.) cinctipes (Provancher)	0	0	0	0	1	0	0	1
Lasioglossum (D.) coreopsis (Robertson)	0	1	0	0	1	1	0	3
Lasioglossum (D.) creberrimum (Smith)	0	1	0	1	0	0	0	2
Lasioglossum (D.) floridanum (Robertson)	39	88	52	0	7	0	4	190
Lasioglossum (D.) hitchensi Gibbs	0	0	0	1	15	5	0	21
Lasioglossum (D.) illinoense (Robertson)	128	67	68	25	25	16	31	360
Lasioglossum (D.) imitatum (Smith)	0	0	0	54	86	48	26	214
Lasioglossum (D.) leviense (Mitchell)	0	0	0	4	18	6	5	33
Lasioglossum (D.) longifrons (Baker)	1	2	1	1	33	14	2	54
Lasioglossum (H.) nelumbonis (Robertson)	0	0	0	12	0	1	0	13
Lasioglossum (D.) nymphale (Smith)	96	113	153	1	1	0	2	366
Lasioglossum (H.) pectorale (Smith)	0	0	0	28	19	41	8	96
Lasioglossum (D.) puteulanum Gibbs	1	1	2	62	75	19	18	178
Lasioglossum (D.) raleighense (Crawford)	0	0	0	0	1	2	0	3

Lasioglossum (D.) reticulatum (Robertson)	46	1	6	211	58	51	166	539
Lasioglossum (H.) sopinci (Crawford)	0	0	1	0	3	1	0	5
Lasioglossum (D.) trigeminum Gibbs	0	0	5	4	28	5	8	50
Lasioglossum (D.) vierecki (Crawford)	0	0	0	0	1	0	11	12
Lasioglossum (D.) weemsi (Mitchell)	0	0	0	21	32	15	19	87
Megachile albitarsis Cresson	0	0	0	1	0	0	3	4
Megachile frugalis Cresson	0	0	0	0	0	0	1	1
Megachile mendica Cresson	0	1	1	2	1	3	1	9
Megachile petulans Cresson	0	0	0	2	2	2	1	7
Megachile pseudobrevis Mitchell	1	4	4	3	0	0	3	15
Megachile texana Cresson	0	0	0	2	4	1	1	8
Melissodes bimaculatus (Lepeletier)	0	0	0	2	4	3	0	9
Melissodes communis Cresson	0	0	0	45	13	6	12	76
Melissodes comptoides Robertson	0	0	0	9	0	0	0	9
Melissodes denticulata Smith	0	0	0	0	0	0	1	1
Melissodes tepaneca Cresson	0	0	0	5	6	4	7	22
Melissodes tinctus LaBerge	0	0	0	0	1	0	0	1
Melitoma taurea (Say)	0	0	0	2	0	0	3	5
Nomada lepida Cresson	0	0	0	1	0	0	0	1
Nomada luteola Olivier	0	0	0	0	1	0	2	3
Osmia inspergens Lovell and Cockerell	0	2	2	0	0	0	0	4
Osmia sandhouseae Mitchell	0	6	0	0	0	0	0	6
Peponapis pruinosa (Say)	0	0	0	0	0	2	0	2
Perdita bishoppi Cockerell	0	2	0	0	0	0	0	2
Perdita consobrina Timberlake	2	2	0	0	0	0	0	4
Perdita georgica Timberlake	0	6	0	0	0	0	0	6
Perdita gerardiae Crawford	4	0	0	0	44	19	93	160
Perdita halictoides Smith	0	0	0	0	0	0	1	1
Perdita octomaculata (Say)	2	0	0	0	0	0	0	2
Pseudopanurgus sp.	0	0	0	0	1	0	0	1
Ptilothrix bombiformis (Cresson)	0	0	0	0	3	5	4	12
Sphecodes atlantis Mitchell	0	0	0	0	0	2	0	2
Sphecodes mandibularis Cresson	0	0	0	0	1	0	0	1
Sphecodes sp.	0	0	0	0	1	0	0	1

Svastra atripes (Cresson)	18	0	3	1	6	0	2	30
Xylocopa micans Lepeletier	0	0	0	0	0	1	0	1
Xylocopa virginica (Linnaeus)	0	0	0	0	3	1	1	5
Total abundance	908	861	920	1260	654	407	859	5869
Total species	18	23	21	45	52	38	42	94

Supplementary Table S2. Results from PERMANOVA for pairwise comparisons of bee community composition between locations

	Comparison	t	p
Red Hills	GWP vs. GWS	1.581	0.0024
	GWP vs. TT	2.523	0.0002
	GWP vs. WT	1.9164	0.0002
	GWS vs. TT	2.8237	0.0004
	GWS vs. WT	2.0853	0.0004
	TT vs. WT	2.396	0.0004
Eglin AFB	E24 vs. F22	2.0378	0.0004
	E24 vs. PATT	2.0372	0.001
	F22 vs. PATT	1.7522	0.0012
